# The effect of preferred music on mental workload and laparoscopic surgical performance in a simulated setting (OPTIMISE): a randomized controlled crossover study

**DOI:** 10.1007/s00464-020-07987-6

**Published:** 2020-10-07

**Authors:** Victor X. Fu, Pim Oomens, Vincent E. E. Kleinrensink, Karel J. Sleurink, Willemijn M. Borst, Pascale E. Wessels, Johan F. Lange, Gert-Jan Kleinrensink, Johannes Jeekel

**Affiliations:** 1 Department of Surgery, Erasmus MC, University Medical Center, Doctor Molewaterplein 40, 3015 GD Rotterdam, The Netherlands; 2 Department of Neuroscience, Erasmus MC, University Medical Center, Doctor Molewaterplein 40, 3015 GD Rotterdam, The Netherlands

**Keywords:** Music, Laparoscopy, Surgical performance, Mental workload, Stress, Heart rate variability

## Abstract

**Background:**

Worldwide, music is commonly played in the operation room. The effect of music on surgical performance reportedly has varying results, while its effect on mental workload and key surgical stressor domains has only sparingly been investigated. Therefore, the aim is to assess the effect of recorded preferred music versus operating room noise on laparoscopic task performance and mental workload in a simulated setting.

**Methods:**

A four-sequence, four-period, two-treatment, randomized controlled crossover study design was used. Medical students, novices to laparoscopy, were eligible for inclusion. Participants were randomly allocated to one of four sequences, which decided the exposure order to music and operation room noise during the four periods. Laparoscopic task performance was assessed through motion analysis with a laparoscopic box simulator. Each period consisted of ten alternating peg transfer tasks. To account for the learning curve, a preparation phase was employed. Mental workload was assessed using the Surgery Task Load Index. This study was registered with the Netherlands Trial Register (NL7961).

**Results:**

From October 29, 2019 until March 12, 2020, 107 participants completed the study, with 97 included for analyzation. Laparoscopic task performance increased significantly during the preparation phase. No significant beneficial effect of music versus operating room noise was observed on time to task completion, path length, speed, or motion smoothness. Music significantly decreased mental workload, reflected by a lower score of the total weighted Surgery Task Load Index in all but one of the six workload dimensions.

**Conclusion:**

Music significantly reduced mental workload overall and of several previously identified key surgical stressor domains, and its use in the operating room is reportedly viewed favorably. Music did not significantly improve laparoscopic task performance of novice laparoscopists in a simulated setting. Although varying results have been reported previously, it seems that surgical experience and task demand are more determinative.

**Electronic supplementary material:**

The online version of this article (10.1007/s00464-020-07987-6) contains supplementary material, which is available to authorized users.

Worldwide, music is commonly played during surgery [1]. Perioperative music has been extensively investigated in adult surgical patients with several beneficial effects [2–4]. However, no definitive conclusion on the effect of music on surgical task performance can currently be drawn due to conflicting study results, inconsistent data reporting methods, and varying study designs in previously published studies [5]. To date, all these studies have been conducted in a simulated setting [5], as surgical performance in a simulated setting correlates to performance during actual real-world surgery and influences postoperative patient outcome [6–9]. It is unclear whether the reported beneficial effects of music on surgical performance are due to an auditory stimulus and not music per se, as all but one [10] of the previous studies used silence as a control [11–18]. Given that high noise level settings are commonly prevalent in the operation room (OR) [19], it could be argued that using silence as a control factor is therefore not appropriate when evaluating the effect of music on surgical performance.

Some surveys have shown that music is well liked by surgical personnel and can improve focus during surgery [1], while others mentioned that it can be distracting and reduce vigilance [20, 21]. Therefore, music during surgery could potentially influence mental workload, which can be defined as the attention that can be directed to a surgical task and the balance of the attention amount used and additionally available when necessary. Increased mental workload is associated with decreased surgical task performance [22]. While perioperative music has a significant beneficial attenuating effect on the physiological stress response in adult surgical patients [3], its effect on mental workload and stress while performing a surgical task has only sparingly been investigated [23].

Laparoscopic surgery requires different skills compared to conventional open surgery due to the use of long instruments and the fulcrum effect, two-dimensional screen visualization which can impair depth perception, and limited tactile feedback [24]. Therefore, simulation using either a box trainer or virtual reality is increasingly used to provide a safe environment for the early learning curve phase. Successfully completing the Fundamentals of Laparoscopic Surgery program is required to become board certified as a general surgeon in the United States [25]. The acquired competencies in a simulated setting seem to be transferable to the real word setting with favorable effects on skill, knowledge, and patient outcome [6, 9, 26]. The purpose of this randomized crossover study is to investigate the effect of participant-selected recorded music versus recorded OR noise on laparoscopic task performance, mental workload, and heart rate variability (HRV) in a simulated setting.

## Materials and methods

This study was approved in September 2019 by the Medical Ethics Committee Erasmus MC (MEC-2019-0537) and prospectively registered with the Netherlands Trial Register (Trial NL7961). The study was performed in accordance with the ethical standards of the Helsinki Declaration of 1975. No study protocol amendments were required. Reporting adhered to the 2010 Consolidated Standard of Reporting Trials (CONSORT) extension for randomized crossover trials [27].

### Study design

**Fig. 1:**
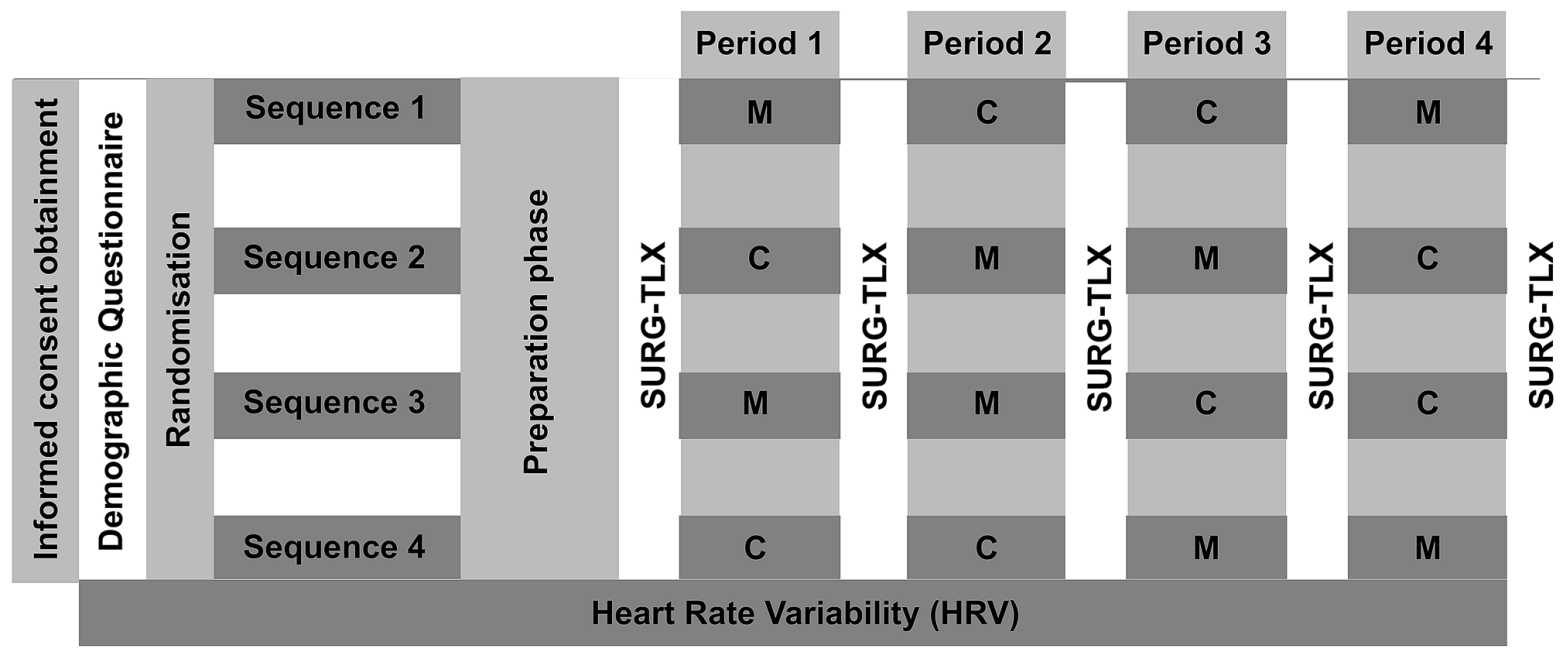
Study procedure overview timeline. Timeline detailing study procedures. Depending on the sequence, consisting of 4 periods, participants were exposed to either music or operation room noise. Preparation phase = 30 alternating peg transfer tasks. Period = 10 alternating peg transfer tasks, *M* Exposure to recorded, participant-selected music, *C*  Exposure to recorded operation room noise, *SURG-TLX* surgery task load index, *HRV * heart rate variability, measured continuously throughout the experiment

A study procedure timeline overview is presented in Fig. 1. A four-sequence, four-period, two-treatment, randomized controlled crossover study design was used to investigate the effects of recorded, participant-selected music versus recorded OR noise on laparoscopic task performance, mental workload, and HRV. Medical students who were novices to laparoscopy and provided written informed consent were eligible for study participation. Severe hearing impairment, visual impairment, physical handicap that impairs laparoscopic task performance, or use of cardiac medication were considered as exclusion criteria. Participants were instructed to bring music they would like to listen to while performing a laparoscopic task and to abstain from alcohol for 12 h prior to the experiment. The 10 min OR noise recording was selected from a list by three authors (VF, PO and JJ) with prior surgical experience in the OR to represent noise during a routine laparoscopic surgical procedure (i.e., no orthopedic drilling noise). Laparoscopic task performance was assessed with a validated, custom-made laparoscopic box simulator using the peg transfer task [23], during which a blue and red peg are moved with a grasper forceps to a predefined location shown on a monitor. This task is part of the Fundamentals of Laparoscopic Surgery program for surgical residents in the United States [28]. Motion data to assess laparoscopic task performance were captured using a Leap Motion Device (LMC, Leap Motion Inc., LM-010), a compact sensor modified and customized for motion analysis, connected to a computer with monitor, and a webcam (Gemini Gembird) functioning as camera with a frame rate of 60 Hz. Motion data were progressed using a custom-made software program (OCRAM technologies) combined with Python version 2.7.

After signing the informed consent form, a chest band was fitted to continuously measure HRV throughout the entire experimental session [29]. A custom demographic questionnaire evaluating music importance and preferences, listening to music while studying, and whether a music instrument is or was played, was filled out. Participants were randomly allocated using the sealed envelope method and a 1:1:1:1 allocation ratio to one of four sequences. Each sequence consisted of a preparation phase followed by two periods of recorded, participant-selected music and two periods recorded OR noise, with the order of exposure decided by the previously mentioned randomization. To account for the learning curve, all participants completed a preparation phase consisting of 30 peg transfer tasks, alternating between the right and left hand (i.e., the first peg transfer was performed using the right hand, the second using the left hand, the third using the right hand again and so on), as it was previously observed that the learning curve flattened after 20 repetitions [23]. During each period, 10 alternating peg transfer tasks were performed while listening to either music or OR noise using noise-canceling headphones (Bose Quietcomfort 35ii). Volume level was adjusted at the start by the participant and was therefore consistent during the entire experiment. The Surgery Task Load Index (SURG-TLX) questionnaire evaluating mental workload was filled out after the preparation phase and each period for a total of five times, which led to a washout period of at least several minutes.

### Outcome parameters

The primary outcome measure was time to task completion, defined as the time in seconds (s) required to complete a 10 peg transfer task period, consisting of alternating peg transfers with the dominant and non-dominant hand. Time to task completion of the peg transfer task is the main score attribute in the Fundamentals of Laparoscopic Surgery program [28]. Furthermore, path length, the total distance traveled in millimeters (mm) by the instrument tip, speed, the ratio of path length and time to task completion (mm/s), and motion smoothness, the normalized jerk or the rate of instrument tip acceleration change (mm/s^3^), were measured. To assess the benefit of the preparation phase, motion analysis of the first 10 peg transfers in this phase was compared to the last 10 peg transfers additionally.

Mental workload was assessed using the SURG-TLX, an in laparoscopic surgery validated, adapted version of the National Aeronautics and Space Administration Task Load Index (NASA-TLX) questionnaire [30]. This weighted questionnaire assesses six dimensions of workload (mental demands, physical demands, temporal demands, task complexity, situation stress and distractions) using a visual analog scale (VAS) and was filled out by all participants after the preparation phase and each period.

Heart rate and HRV, defined as the variation in time between each heartbeat (NN), were continuously measured from the preparation phase start until experiment end using the commercially available, validated BM-CS5EU wireless chest band (BM innovations, Acentas GmbH) [29]. Short-term HRV measurements [31], lasting approximately five minute during each of the four periods as well as the first five and last five minutes of the preparation phase, were analyzed (ATS 2.4.6., BM Innovations). HRV can represent the physiological state of autonomic nervous system activity and has been used to assess mental strain in surgeons during laparoscopic task performance [32]. A lower HRV implicates dominance by the sympathetic nervous system and has been regarded as higher mental strain. HRV quantification was presented using the time-domain variable standard deviation of all NN intervals (SDNN) in milliseconds (ms).

### Blinding and data analysis

Obviously, the participants in this experiment could not be blinded. Headphones were employed partly to blind the research assistant overseeing the experiment. However, as participants brought their preferred music using different devices, transferring music to the laptop which contained the OR noise recording in order to be played during the experiment was impractical. Therefore, the music intervention was directly played while the headphones were attached to the participant’s phone or music player. Although the research assistant was separated from the participant by an opaque screen during the experiment in order to reduce any influence to the fullest extent, the assistant was not considered to be blinded. All questionnaires were filled out using a secure, computerized questionnaire by the participants themselves and were therefore not administered by the research assistant. Heart rate and HRV data were processed through a validated software program. Motion data analysis was computerized using a software script validated in previous studies [23], while the person responsible for data retrieval and preparing it for analysis was blinded to the allocation sequence. All data were only analyzed after the last participant had completed the experiment.

Data were statistically analyzed using the IBM Statistical Package for the Social Sciences (SPSS) version 24.0. Data were presented as mean and standard deviation (SD) if data were normally distributed, and median and interquartile range (IQR) if not. Normality of data was assessed using the Kolmogorov–Smirnov test and visually in Q–Q plots. Continuous variables were compared using a paired-samples *T* test or Wilcoxon signed rank test, as appropriate. Within subject differences were presented by subtracting the control group from the intervention group. Categorical variables were presented as absolute number and percentage. Two tailed testing was used with statistical significance inferred at *p* < 0.05.

### Sample size calculation

Based on our previous study using the same laparoscopic box simulator [23], an effect size of 0.3 was deemed clinically relevant. With alpha set at 0.05, power of 0.80 and two-sided dependent testing, 90 participants would be required. Given that there were four randomization sequences, we chose to set the sample size at 92 participants to allow for equal distribution among the sequences. Taking into account a 10 percentage exclusion rate, total sample size was set at 104 participants.

## Results

**Fig. 2:**
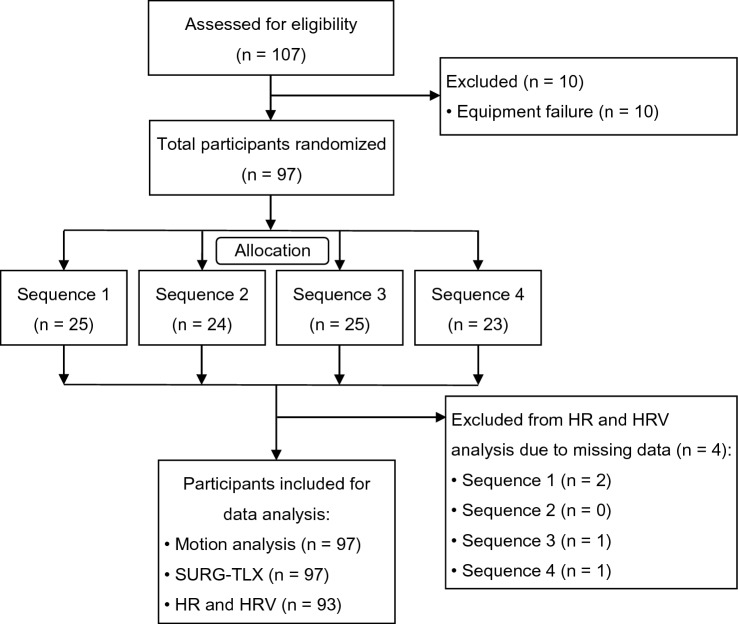
CONSORT flow diagram. Inclusion flowchart. *N*  number of participants, *SURG-TLX* surgery task load index, *HR* heart rate, *HRV* heart rate variability

From October 29, 2019 until March 12, 2020, 107 participants were recruited. Ten participants were excluded because of equipment failure at the start of the study. Motion analysis and mental workload assessment using the SURG-TLX was performed of all 97 participants who completed the study. Due to missing data, heart rate and HRV analysis was performed of 93 participants (Fig. 2).

### Demographic characteristics

An overview of demographic characteristics of the full cohort (*n* = 97) can be found in Table 1. Median age was 20 (IQR 18 to 21), with the majority of the medical students being in their first three years of study (77%), right-handed (85%), and female (57%). A little over half of participants (54%) had experience with a musical instrument, with 31 (32%) currently playing and 21 (22%) previously playing an instrument. Music was deemed important in daily life with a median numeric rating scale (NRS) of 8 (IQR 7 to 8), with 68 (70%) participants listening to music while studying. Favorite genres while studying were classical (20%) and pop (16%), while 18% specified music that could not be classified under commonly described genres. Top music genres chosen for this experiment were pop (47%), classical (21%), and hip hop (9.3%) (Online Appendix A).

**Table 1:** Demographic characteristics

Characteristic	Full cohort (*n* = 97)
Age (years)	20 (18–21)
Sex	
Male	42 (43.3%)
Female	55 (56.7%)
Dexterity	
Right	82 (84.5%)
Left	14 (14.4%)
Bimanual	1 (1.0%)
Year of medical school	
1	34 (35.1%)
2	20 (20.6%)
3	21 (21.6%)
4	16 (16.5%)
5	1 (1.0%)
6	5 (5.2%)
Importance of music (NRS)	8 (7–8)
Listens to music while studying	
Yes	68 (70.1%)
No	29 (29.9%)
Top three favorite music genre while studying	
1	Classical (19.6%)
2	Other (17.5%)
3	Pop (15.5%)
Plays a musical instrument	
Yes	31 (32.0%)
Used to	21 (21.6%)
No	45 (46.4%)
Top three chosen music genre for OPTIMISE study	
1	Pop (42.3%)
2	Classical (19.6%)
3	Hip hop (9.3%)
Sequence randomization	
1	25 (25.8%)
2	24 (24.7%)
3	25 (25.8%)
4	23 (23.7%)

Demographic characteristics of OPTIMISE study population. For categorical variables, absolute number (percentage of study population) is presented. For continuous variables, median (interquartile range) is presented

*n* number of participants, *NRS* numeric rating scale

### Laparoscopic task performance

Laparoscopic task performance improved during the preparation phase (Table 2), with time to task completion of the last 10 alternating peg transfer tasks being significantly faster compared to the first 10 tasks (median 250 s [IQR 218 to 327] versus 433 s [335 to 532], *p* < 0.001). A significant reduction in path length of the last 10 compared to the first 10 tasks during the preparation phase (8375 mm [6107 to 12397] versus 12810 mm [8813 to 18168], *p* < 0.001) and improved motion smoothness in the form of normalized jerk was also observed (236596 mm/s^3^ [102441 to 471534] versus 857493 mm/s^3^ [407460 to 1833467], *p* < 0.001).

**Table 2:** Preparation phase

Motion analysis (*n* = 97)	First 10 peg transfer tasks	Last 10 peg transfer tasks	Within subject differences	*p*
Time to task completion, median (IQR), s	433 (335 to 532)	250 (218 to 327)	163 (65.9 to 266)	0.000
Path length, median (IQR), mm	12810 (8813 to 18168)	8375 (6107 to 12397)	4018 (761.8 to 7354)	0.000
Average speed, median (IQR), mm/s	445 (321 to 540)	461 (325 to 561)	− 6.21 (− 81.7 to 72.9)	0.544
Normalized jerk, median (IQR), mm/s^3^	857493 (407460 to 1833467)	236596 (102441 to 471534)	493611 (97813 to 1334364)	0.000

Heart rate and heart rate variability (*n* = 93)	First 5 min	Last 5 min	Within subject differences	*P*
Heart rate, median (IQR), bpm	87 (75 to 101)	91 (80 to 106)	− 4.0 (− 8.0 to − 0.50)	0.000
Heart rate variability, median (IQR), SDNN	52 (40 to 71)	48 (38 to 63)	7.0 (− 1.5 to 13)	0.000

Learning curve overview reflected by comparing the motion analysis of the last 10 to the first 10 peg transfer tasks during the preparation phase, which consisted of 30 alternating peg transfer tasks in total. Data presented as median (interquartile range)

*n* number of participants, *IQR* interquartile range, *s* seconds, *mm* millimeters, *bpm* beats per minutes, *SDNN* standard deviation of the all NN intervals, representing a median of HRV variability

No statistically significant difference in laparoscopic task performance parameters were observed during exposure to the different auditory stimuli (Table 3). Time to task completion was not statistically significantly faster while listening to music compared to OR noise (210 s [191 to 262] versus 221 s [188 to 257], *p* = 0.518) and path length was not reduced (7606 mm [5725 to 9182] versus 7462 mm [5833 to 8952], *p* = 0.434). Speed did not differ (434 mm/s [321 to 552] versus 436 mm/s [324 to 556], *p* = 0.758), nor did motion smoothness in the form of normalized jerk (180687 mm/s^3^ [83581 to 281566] versus 171957 mm/s^3^ [95905 to 316327], *p* = 0.125). Additionally, there was no significant difference by music compared to OR noise in laparoscopic task performance parameters of the dominant and non-dominant hand, when these were assessed separately from each other. When assessing the participants who preferred to listen to music when studying (n = 68) as a separate group, no significant difference was observed. No difference was observed when taking experience with playing a musical instrument into account, or gender (Table 4).

**Table 3:** Main study results

	Music	Control	Within subject differences	*p*
Motion analysis (*n* = 97)				
Time to task completion, median (IQR), s	210 (191 to 262)	221 (188 to 257)	0.60 (− 25.3 to 18.8)	0.518
Path length, median (IQR), mm	7606 (5725 to 9182)	7462 (5833 to 8952)	184.3 (− 853.0 to 1219)	0.434
Average speed, median (IQR), mm/s	434 (321 to 552)	436 (324 to 556)	0.83 (− 50.8 to 55.6)	0.758
Normalized jerk, median (IQR), mm/s^3^	180687 (83581 to 281566)	171957 (95905 to 316327)	− 9259 (− 78294 to 45858)	0.125
Mental workload (*n* = 97)				
SURG-TLX, median (IQR), VAS 0–100	27.0 (17.3 to 38.3)	33.7 (21.2 to 43.3)	− 3.50 (− 9.08 to 1.75)	0.000
Mental demands, median (IQR), VAS 0–100	25.0 (15.0 to 37.5)	30.0 (20.0 to 50.0)	− 5.00 (− 12.5 to 0.00)	0.000
Physical demands, median (IQR), VAS 0–100	20.0 (12.5 to 32.5)	22.5 (12.5 to 31.3)	0.00 (− 5.00 to 2.50)	0.012
Temporal demands, median (IQR), VAS 0–100	35.0 (20.0 to 47.5)	37.5 (22.5 to 50.0)	− 2.50 (− 12.5 to 5.00)	0.010
Task complexity, median (IQR), VAS 0–100	25.0 (12.5 to 42.5)	25.0 (15.0 to 43.8)	0.00 (− 5.00 to 2.50)	0.471
Situational stress, median (IQR), VAS 0–100	17.5 (10.0 to 30.0)	25.0 (12.5 to 40.0)	− 2.50 (− 10.0 to 0.00)	0.000
Distractions, median (IQR), VAS 0–100	15.0 (10.0 to 30.0)	32.5 (20.0 to 47.5)	− 10.0 (− 27.5 to 0.00)	0.000
Heart rate and heart rate variability (*n* = 93)				
Heart rate, median (IQR), bpm	88 (78 to 102)	87 (77 to 102)	1.0 (− 1.3 to 2.0)	0.046
Heart rate variability, median (IQR), SDNN	49 (40 to 63)	52 (41 to 68)	− 2.5 (− 6.8 to 2.8)	0.015

Overview of main study results with data presented as median (interquartile range)

*n*  number of participants, *IQR* interquartile range, *s* seconds, *mm* millimeters, *bpm* beats per minutes, *SDNN* standard deviation of the all NN intervals, representing a median of HRV variability

**Table 4:** Additional motion analysis

	Music	Control	Within subject differences	*p*
Motion analysis (*n* = 96)				
Time to task completion, median (IQR), s				
Dominant hand	102 (85.9 to 121)	104 (89.2 to 122)	− 2.91 (− 17.6 to 10.9)	0.271
Non-dominant hand	111 (91.2 to 136)	110 (94.0 to 140)	− 0.67 (− 16.5 to 18.1)	0.953
Path length, median (IQR), mm				
Dominant hand	3729 (2109 to 5300)	3685 (2446 to 5042)	− 16.31 (− 613.5 to 609.3)	0.852
Non-dominant hand	3537 (2285 to 4594)	3415 (2187 to 4269)	10.94 (− 431.9 to 673.5)	0.494
Average speed, median (IQR), mm/s				
Dominant hand	224 (148 to 314)	221 (153 to 326)	− 3.02 (− 38.7 to 37.5)	0.924
Non-dominant hand	199 (148 to 239)	200 (143 to 243)	2.02 (− 19.2 to 18.1)	0.648
Normalized jerk, median (IQR), mm/s^3^				
Dominant hand	95520 (34549 to 179223)	91289 (38076 to 214895)	− 5856 (− 69945 to 37479)	0.116
Non-dominant hand	48433 (27678 to 106052)	49010 (28656 to 94344)	− 1642 (− 30473 to 20571)	0.614
Motion analysis (*n* = 97)				
Time to task completion, median (IQR), s				
Listens music while studying (*n* = 68)	208 (189 to 254)	222 (187 to 253)	− 1.85 (− 30.2 to 12.4)	0.248
No music while studying (*n* = 29)	222 (200 to 271)	211 (188 to 262)	5.18 (− 20.1 to 34.8)	0.381
Time to task completion, median (IQR), s				
Plays or played instrument (*n* = 52)	205 (184 to 253)	216 (184 to 243)	− 1.85 (− 22.0 to 10.7)	0.377
Never played instrument (*n* = 45)	216 (201 to 267)	230 (194 to 270)	5.03 (− 28.1 to 29.8)	0.861
Time to task completion, median (IQR), s				
Male (*n* = 42)	206 (183 to 262)	211 (180 to 244)	− 7.72 (− 23.2 to 26.3)	0.722
Female (*n* = 55)	214 (197 to 264)	231 (200 to 264)	2.34 (− 26.5 to 16.2)	0.861

Effect of participant-selected music versus recorded operation room noise on laparoscopic task performance by the dominant and non-dominant hand (one participant was ambidextrous), and the influence of listening to music while studying or playing a musical instrument on time to task completion

*n* number of participants, *IQR* interquartile range, *s* seconds, *mm * millimeters

### Mental workload, heart rate, and HRV

A significant beneficial effect of music was observed on mental workload as the weighted SURG-TLX score was lower (27.0 [17.3 to 38.3] versus 33.7 [21.2 to 43.3], *p* < 0.001). This was also reflected in all but one of the SURG-TLX dimensions (Table 3, Online Appendix B). Mental demands (25.0 [15.0 to 37.5] versus 30.0 [20.0 to 50.0], *p* < 0.001), physical demands (20.0 [12.5 to 32.5] versus 22.5 [12.5 to 31.3], *p* = 0.012), temporal demands (35.0 [20.0 to 47.5] versus 37.5 [22.5 to 50], *p* = 0.010), situational stress (17.5 [10.0 to 30.0] versus 25.0 [12.5 to 40.0], *p* < 0.001), and distractions (15.0 [10.0 to 30.0] versus 32.5 [20.0 to 47.5], *p* < 0.001) were all significantly lower when exposed to music. Only in the task complexity dimension, no significant difference was observed (25.0 [12.5 to 42.5] versus 25.0 [15.0 to 43.8], *p* = 0.471).

In four participants (4.1%), heart rate and HRV data were not registered, and data were therefore analyzed of 93 participants. None of the included participants had known cardiac diseases or arrhythmias or used any cardiac medication. Median duration of HRV measurement was 4.25 min [3.59 to 5.11] over the experiment (93 measurements per period) as a whole. Of the 372 total heart rate and HRV measurements, the measurement duration of 173 (47%) were at least 4.5 min or more, 166 (45%) were between 3.5 and 4.5 min, and 33 were below 3.5 min (8.9%). Heart rate during the last 5 min of the preparation phase was statistically significantly increased compared to the first 5 min (91 [80 to 106] versus 87 [75 to 101], *p* < 0.001), while HRV was statistically significantly lower (48 [38 to 63] versus 52 [40 to 71], *p* < 0.001) (Table 2). During the experiment, heart rate was statistically significantly higher while exposed to music (88 [78 to 102] versus 87 [77 to 102], *p* = 0.046). HRV was statistically significantly lower while exposed to music (49 [40 to 63] versus 52 [41 to 68], *p* = 0.015) (Table 3).

No correlation was present between HRV and mental workload assessed using the SURG-TLX (Spearman’s rho 0.060, *p* = 0.565), nor between heart rate and SURG-TLX (Spearman’s rho -0.022, *p* = 0.836).

## Discussion

This randomized controlled crossover study with the largest sample size to date assessed the effect of participant-selected recorded music on laparoscopic task performance and mental workload in a simulated setting. No statistically significant beneficial effect of participant-selected music was observed regarding laparoscopic task performance while compared to OR noise in novice laparoscopists. Previous studies, all performed in a simulated setting, reported varying results [5]. Two studies with a similar study design and comparable tasks by the same lead author evaluated the effect of music on laparoscopic task performance. A beneficial effect on task accuracy in expert surgeons was observed [11], but not in junior residents with no previous laparoscopic experience [12]. No beneficial effects were observed in junior novice surgeons asked to perform part of a laparoscopic cholecystectomy [15], nor in 12 surgeons with varying experience placing laparoscopic knots [10]. Although considered a basic skill, laparoscopic knot tying is reportedly the most difficult laparoscopic skill to master [33, 34]. In aforementioned studies, preselected music by the research team was used. A positive trend between likability of the music and a beneficial effect was noted [15]. In practice, it seems less likely that surgeons would listen to music that they do not prefer. Surgeons did choose the music played in the OR in a majority of cases [35–37]. Hence, participant-selected preferred music was used which we believe to be more clinically relevant. Recently, we observed a significant beneficial effect on time to task completion (4.68%, *p* = 0.037) and path length (6.35%, *p* = 0.019) of participant-selected music versus silence in 60 medical students in our previous study. Surgical experience level was comparable, as they were also novices of laparoscopy, and a similar study setup was employed, although the modified peg transfer task was only performed 5 times with solely the dominant hand [23]. It could be argued that the different results compared to this study on task performance can partly be attributed due to a more demanding task, with a higher SURG-TLX and heart rate in this study [23]. Therefore, given the previously mentioned studies, it might be possible that depending on experience and task complexity, music could be beneficial when the surgical task is considered to be relatively easy and manageable, but that this effect disappears when the motor task is more difficult and increasingly demanding on mental workload.

An important component during laparoscopic surgery besides motor task execution and performance is the cognitive decision making to determine which motor steps should be executed. Reducing mental workload, often reported as stress by the surgeon, will leave more mental resource capacity for both components [38]. Indeed, laparoscopic task performance has been correlated to stress experienced by the surgeon [39], with identified key stressors in the form of time pressure, noise and distractions impairing dexterity, and increasing error rate [40]. Mental workload assessed using the SURG-TLX questionnaire was significantly reduced by music, which was especially profound in the domains mental demands (within subject difference -5.0, *p* = 0.000) and distractions (within subject difference -10.0, *p* = 0.000), while reflected to a slightly lower but still significant degree in temporal demands (within subject difference -2.5, *p* = 0.010). While secondary outcome measure results should always be interpreted with caution, these findings mimic our previous study which also observed a beneficial effect by music on mental workload during laparoscopic task performance [23]. Previous surveys also observed favorable responses in general towards the use of music by surgeons, especially in regard to stress [36, 41, 42]. Although reporting bias cannot be entirely ruled out, the SURG-TLX follows the trend of objective parameters like salivary cortisol levels [43]. HRV seems to be an adequate method to assess mental surgical stress as well [44]. While heart rate was statistically significantly higher (within subject difference 1.0 bpm, *p* = 0.046) and HRV lower (within subject difference − 2.5, *p* = 0.015) in the music group, the absolute difference observed cannot be considered clinically relevant. It was expected that each period in this experiment would allow for short-term HRV analysis (nominal 5 min duration), but 54% of HRV measurements lasted 4.5 min or less. Given that the validity of ultra short-term HRV analysis has been questioned [31], as well as the lack of correlation with mental workload in this study, interpretation of these results as a reflection of mental strain in our study should be done with caution.

Major strong points of this study was the largest sample size to date and the rigorous study design, which reduces a potential carryover effect [45]. While computerized randomization would preferably be used, we considered non-random allocation risk to be minimal. All participants acted as their own control. The research assistant overseeing the experiment execution had no incentive to influence allocation as they had no information on the participant during the experiment, given that all questionnaires were filled out using secure computerized questionnaire. These data were only revealed and analyzed after all inclusions had been completed. The envelope deciding allocation sequence was chosen before the preparation phase, preventing any potential influence of this phase on the allocation sequence. A maximum envelope number per sequence based on the sample size calculation assured equal allocation. A previously validated, custom-made laparoscopic box trainer was used [23], with real surgical instruments and pegs allowing for realistic tactile sense and haptic feedback that is not provided by all virtual reality simulators. To get acquainted with the box trainer and eliminate the learning curve as the foremost potential biasing factor, a preparation phase was incorporated. The number of peg transfer tasks necessary for this was based on a previously conducted study with the same box trainer [23]. Its success is evident through the fact that time to task completion rapidly decreased in the preparation phase, while it stayed almost consistent during the experiment (for either treatment factor). Since previous studies did not employ a preparation phase, it is difficult to ascertain whether the previously reported effects partly reflect the learning curve. Moreover, participant-selected instead of researcher-selected recorded music was used and the volume adjusted by the participants themselves to more accurately represent the real-world setting, while recorded OR noise acted as a control instead of silence in order to account for auditory stimulation as a factor. Nonetheless, several limitations can still be observed. The peg transfer task was chosen, which does not require surgical knowledge that could potentially influence task performance. However, this task with an average observed duration of approximately 3.5 min per period takes significantly shorter than any surgical procedure. Still, earlier studies reported that even relatively simple, short-lasting tasks and drills like these can improve relevant laparoscopic surgical tasks and should therefore not be disregarded [46, 47]. We chose to perform the study in medical students who were inexperienced with laparoscopy in order to reduce potential previous experience influencing laparoscopic task performance. Studies evaluating noise in the OR found higher subjective distraction levels in assisting surgeons with less experience compared to the main, more experienced surgeons [48], while the negative impact on clinical reasoning was lower when anesthesiological residents were more experienced [49]. It has been theorized that more experienced surgeons can block out noise and music more effectively [10], theoretically decreasing potential effect size and increasing the required number of participants. It would have been impractical therefore to try to investigate the effects of music using more experienced residents or surgeons in such large numbers without the present data and our recently published study [23]. Finally, a major factor affecting teamwork in the OR is communication, with a considerable percentage of surgical errors involving communication between surgical personnel [50]. This factor could not be evaluated. These limitations make extrapolation of the observed results to the real-world setting less appropriate, limiting conclusions to a simulated setting.

Although varying results regarding the effects of music on laparoscopic task performance have been reported, it seems that surgical experience and task demand can be more determinative. Future studies should take these factors into account and evaluate surgeons with different experience levels in a more lifelike setting. While several studies evaluated the effect of music on laparoscopic task performance through short-lasting laparoscopic and surgical tasks to date [5], important elements like simulated surgical procedures, communication, and performance of the entire OR team have only sparingly been investigated [51, 52]. Auditory intervention should preferably consist of music combined with OR noise versus OR noise through speakers, with music chosen by both the surgeon and OR team. Music did significantly reduce mental workload and several previously identified key stressors of surgery, and its use in the operating theater is reportedly viewed favorably. Higher perceived stress is associated with a decreased HRV even throughout the night, indicative of a protracted recovery time [44]. As music can attenuate the stress response to surgery in patients undergoing surgery, future research should incorporate its effect on mental workload through HRV with attention to recovery from surgical task performance as well.

## Conclusion

In this four-sequence, four-period, two-treatment, randomized controlled crossover study of 97 laparoscopy novices, recorded preferred music significantly reduced mental workload overall and in key surgical stressor domains during laparoscopic task performance in a simulated setting when compared to OR noise, but no beneficial effect on task performance itself was observed.

## Electronic supplementary material

Below is the link to the electronic supplementary material.

Supplementary file1 Appendix A. Music genres Figure summarizing the music genre chosen for the experiment by the 97 participants (left bar), as well as the preferred genre while studying by 68 participants who like to listen to music while studying (right bar). Data presented are absolute numbers. (TIF 603 kb)

Supplementary file2 Appendix B. Mental workload Effect of participant-selected music and operation room noise on mental workload (Surgery Task Load Index (Surg-TLX)) during laparoscopic task performance. Data is presented as median and interquartile range. Of the three paired bars of the total weighted SURG-TLX and its workload dimensions, the left bar reflects absolute score during participant-selected music exposure, the middle bar during operation room noise exposure, and the right bar the within subject difference. All were statistically significant in favor of music (p<0.05, marked with *), except the dimension task complexity. (TIF 238 kb)

## Abbreviations

bpm: Beats per minutes
CONSORT: Consolidated standard of reporting trials
ECG: Electrocardiography
HRV: Heart rate variability
IQR: Interquartile range
mm: Millimeter
ms: Millisecond
NASA-TLX: National aeronautics and space administration task load index
NN: Interval between two heartbeats
NRS: Numeric rating scale
OR: Operating room
RCT: Randomized controlled trial
s: Second
SD: Standard deviation
SDNN: Standard deviation of all NN intervals, representing HRV median variability
SPSS: Statistical package for the social sciences
SURG-TLX: Surgery task load index
VAS: Visual analog scale
