# Supplementary figures and images for: The effect of preferred music on mental workload and laparoscopic surgical performance in a simulated setting (OPTIMISE): a randomized controlled crossover study
Source: Surg Endosc. 2020 Oct 7;35(9):5051–61. doi: 10.1007/s00464-020-07987-6 (PMC8346395; doi:10.1007/s00464-020-07987-6)

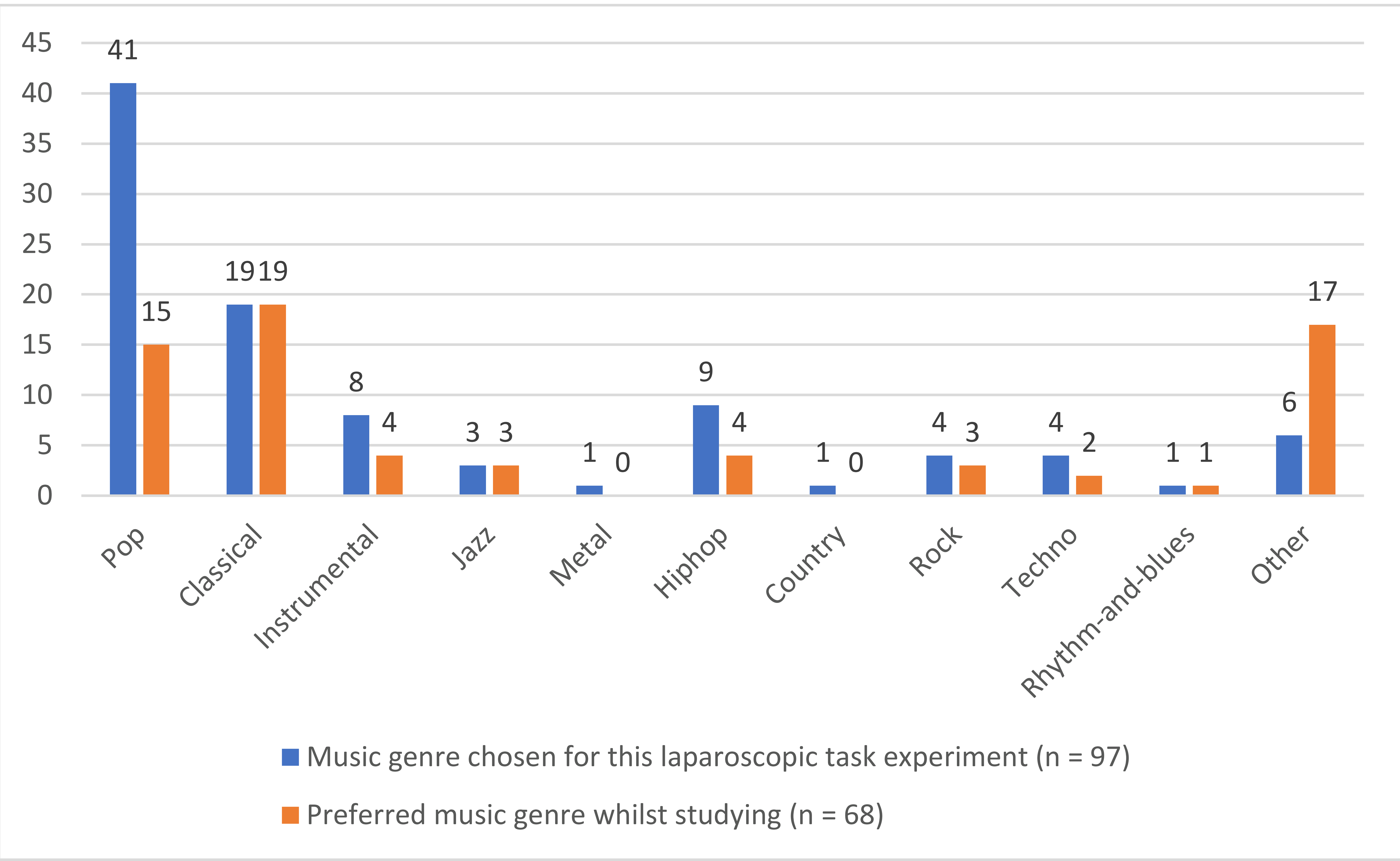

Supplement: Supplementary file 1 — Supplementary file1 Appendix A. Music genres Figure summarizing the music genre chosen for the experiment by the 97 participants (left bar), as well as the preferred genre while studying by 68 participants who like to listen to music while studying (right bar). Data presented are absolute numbers. (TIF 603 kb) [file 464_2020_7987_MOESM1_ESM.tif]

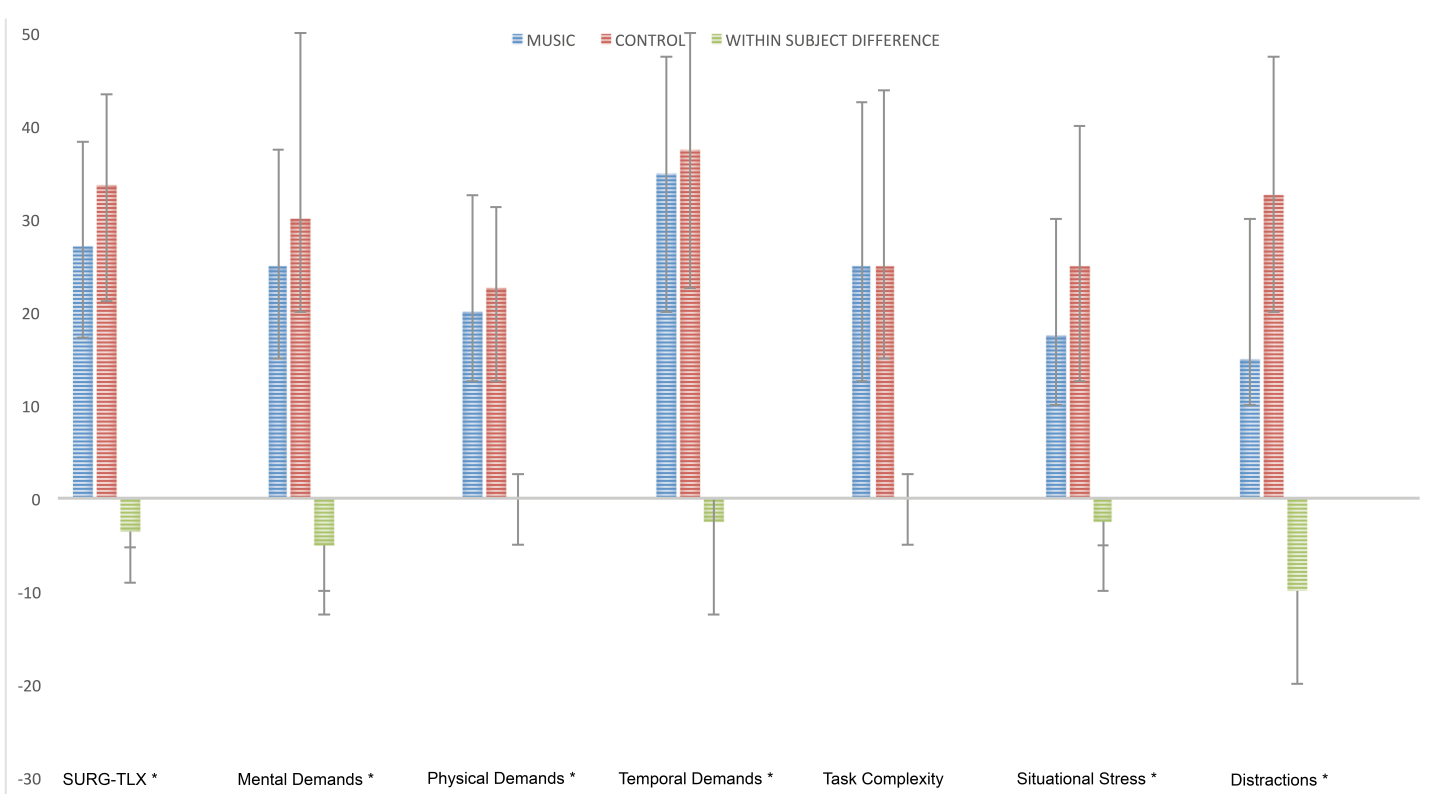

Supplement: Supplementary file 2 — Supplementary file2 Appendix B. Mental workload Effect of participant-selected music and operation room noise on mental workload (Surgery Task Load Index (Surg-TLX)) during laparoscopic task performance. Data is presented as median and interquartile range. Of the three paired bars of the total weighted SURG-TLX and its workload dimensions, the left bar reflects absolute score during participant-selected music exposure, the middle bar during operation room noise exposure, and the right bar the within subject difference. All were statistically significant in favor of music (p<0.05, marked with *), except the dimension task complexity. (TIF 238 kb) [file 464_2020_7987_MOESM2_ESM.tif]
